# Metagenome-Assembled Genomes from Appalachian Acid Mine Drainage Sites

**DOI:** 10.1128/mra.01280-22

**Published:** 2023-04-04

**Authors:** Caden Williams, Jennifer Macalady, Christen Grettenberger

**Affiliations:** a Department of Earth and Planetary Sciences, University of California, Davis, California, USA; b Department of Geosciences, Pennsylvania State University, University Park, Pennsylvania, USA; c Department of Ecotoxicology, University of California, Davis, California, USA; Portland State University

## Abstract

Here, we report 7 metagenome-assembled genomes (MAGs) isolated from acid mine drainage sites in the eastern United States. Three genomes are *Archaea*, including two from the phylum *Thermoproteota* and one from *Euryarchaeota*. Four genomes are bacterial, with one from the phylum *Candidatus* Eremiobacteraeota (formerly WPS-2), one from *Acidimicrobiales* (*Actinobacteria*), and two from *Gallionellaceae* (*Proteobacteria*).

## ANNOUNCEMENT

Acid mine drainage (AMD) sites are unique environments due to their low pH and high metal concentrations. Although many organisms are intolerant of these conditions, bacterial and archaeal acidophiles inhabit AMD sites (1–5). AMD is a major environmental issue and creates extreme ecosystems where microbial communities drive biogeochemical cycling (3, 6–10). For example, some species oxidize metal sulfides or dissolved metal species, facilitating acidification (9, 11–14). However, few genomes are available from AMD environments, especially from newly discovered lineages. This genome report provides genome sequences from 7 AMD populations which provide insight into the microbial activity and diversity in AMD.

Samples were collected from four AMD sites across the Appalachian region, including Porcupine Run in Pennsylvania (41.34378 N, 78.37827 W), Martin Run in West Virginia (39.550828 N, 79.639311 W), and Maxine Mine Geological Overburden Pile (GOB) (33.57420 N, 87.15634 W) and Simpson (33.56417 N, 87.17316 W) in Alabama. Approximately 3 grams of surface sediment was collected in a sterile tube, preserved with 15 mL of RNALater, transported on ice, and stored at −20°C. DNA was extracted using a PowerBiofilm DNA extraction kit (MoBio, Carlsbad, USA). Each sample was extracted twice with a 2- and 4-min vortex adaptor bead beating time, and the products were pooled. Libraries were prepared using the Nextera XT library preparation kit following the manufacturer’s instructions. Paired-end 2 × 150-bp sequencing was performed using an Illumina HiSeq 2500 instrument. Dissolved oxygen, pH, and oxidation reduction potential (ORP) were measured using a Hach HQ40d portable meter. Ferrous iron ([Fe2+]) was measured using a Hach DR 2700 spectrophotometer (Hach, Loveland, USA) (Table 1).

**TABLE 1:** Summary of site geochemistry, metagenome statistics, and MAG properties

Site	Site geochemistry				Metagenome statistics				Genome statistics									
	pH	ORP	[Fe] (mg/L)	Dissolved oxygen (mg/L)	Accession no.	No. of quality-controlled reads	No. of contigs	*N*_50_ (bp)	Accession no.	Genome name	Classification	Total length (Mb)	GC (%) content	No. of contigs	Completeness (%)	Contamination (%)	Coverage (×)	No. of protein-coding genes
Porcupine Run	4.2	261.1	36.5	7.2	SAMN32007064	3,671,780	2,946	7,242	SAMN32097021	Porcupine6	*Bacteria*; *Candidatus* Eremiobacteraeota; *Eremiobacteria*; *Baltobacterales*; *Ca.* Baltobacteraceae; *Ca.* Rubrimentiphilum; JABEUX01 sp01304 4185	2.41	62.3	184	94.5	2.01	16.2	2,433
									SAMN32097022	Porcupine7	*Bacteria*; *Actinobacteriota*; *Acidimicrobiia*; *Acidimicrobiales*; *Acidimicrobiaceae*; *RAAP-2*	2.48	63.1	146	98.29	2.99	24.0	2,422
Martin Run	3.8	477	7.8	9.3	SAMN32007066	7,611,764	700	7,575	SAMN32097023	Martin2	*Archaea*; *Thermoproteota*; *Nitrososphaeria*; *Nitrososphaerales*; *Ca.* Nitrosotalea	1.7	37.9	97	99.51	0.97	11.7	2,016
Maxine Mine GOB	4.0	134.5	199	0.4	SAMN32007065	6,021,450	2,056	5,836	SAMN32097024	Maxine3	*Archaea*; *Thermoproteota*; *Nitrososphaeria*; *Nitrososphaerales*; CADDZS01; *CADDZS01*	2.78	44.3	237	94.66	3.16	17.0	3,119
									SAMN32097025	Maxine5	*Archaea*; *Euryarchaeota*; *Thermoplasmata*; *Methanomassiliicoccales*	1.76	51.2	131	95.81	0.8	12.1	1,767
Simpson	7.3	−84.7	1.3	0.7	SAMN32007067	1,675,384	1,071	9,715	SAMN32097019	Simpson2	*Bacteria*; *Proteobacteria*; *Gammaproteobacteria*; *Burkholderiales*; *Gallionellaceae*; *Gallionella*	1.97	51.4	101	97.14	0.99	11.3	1,985
									SAMN32097020	Simpson3	*Bacteria*; *Proteobacteria*; *Gammaproteobacteria*; *Burkholderiales*; *Gallionellaceae*; *Sideroxydans*	2.21	55.2	241	94.5	2.76	8.7x	2,260

Data processing and analysis were performed in KBase using default parameters (15). Trimmomatic (v.0.36) removed adaptors and low-quality regions (16). The quality of the trimmed data was checked using FastQC (v.0.11.9) (17). Reads were assembled using MEGAHIT (v.1.2.9) (18). Contigs were binned with MetaBAT2 contig binning (v.1.7) (19). Bins were filtered by quality using CheckM (v.1.0.18) for completeness of ≥90% and contamination of ≤5% (20). Bins were classified with GTDB-Tk (v.1.7.0) which assigns genomes to domains using marker genes and places genomes into a reference tree using domain-specific markers (21). Genomes were annotated with Prokka (v.1.14.5) (22). Genome coverage was calculated by mapping the trimmed reads to the MAG using Bowtie 2 (v.2.3.2) (23).

We recovered 7 MAGs. The MAGs were 94.7 to 99.5% complete with 0.8 to 3.2% contamination and contained 1.7 to 2.8 Mbp each (Table 1). GC content was 37.9 to 63.1%. Genomes were contained in 97 to 241 contigs. Three genomes were archaeal, including two from the order *Nitrososphaerales* and one from *Thermoplasmatales*. Four genomes were bacterial, including one from the family *Candidatus* Baltobacteraceae within the recently described phylum *Candidatus* Eremiobacteraeota (formerly WPS-2), one from the actinobacterial order *Acidimicrobiales*, and two from the family *Gallionellaceae*.

### Data availability.

Sequence files are available under BioProject PRJNA907387. Unassembled metagenomic sequences are available under accessions SRX18535480 to SRX18535477, and draft genomes are available under accessions SAMN32097019 to SAMN32097025.
